# Adaptive driver following model that integrates perception process and driving behavior

**DOI:** 10.1038/s41598-022-25952-2

**Published:** 2022-12-14

**Authors:** Changhao Piao, Kexin Li, Qifan Yu, Junren Shi

**Affiliations:** 1 College of Automation, Chongqing University of Posts and Telecommunications, Chongqing, 400000 China; 2 R&D Department, Xi’an Zhongxingxin Software Co., Ltd., Xi’an, 7101140 China

**Keywords:** Electrical and electronic engineering, Mechanical engineering

## Abstract

In order to meet the personalized needs of Chinese intelligent vehicles and improve the satisfaction and acceptance of human–computer interaction and collaboration in domestic intelligent vehicles. In this paper, we design an adaptive longitudinal following model that integrates the perceptual perturbation process and driver characteristics for simulating driver following behavior and studying the variability of driver following behavior. Firstly, for the independence and randomness of driver perception process, a set of random variables conforming to Wiener process is introduced to simulate the perception process of speed and following distance of the vehicle in front; secondly, for the characteristic differences of different drivers' following behavior, a driver characteristic parameter identification algorithm is designed to identify the expected collision time distance and following distance parameters of different drivers, and the identified parameters will be used for Again, a sliding mode control system based on fuzzy switching gain adjustment is designed to simulate the driver following control system. The results show that the designed following model recognizes the driver's characteristics well and can better simulate the driver's following behavior, and the following index is relatively improved by 80%.

## Introduction

Data from the 2018 World Health Organization Global Status Report on Road Safety^1^ show that traffic accidents due to driver factors account for many road accidents. The perceptual perturbation caused by the objective environment and the different driving behaviors of different drivers under this condition are the key factors causing accidents. Effectively identifying drivers' behavioral characteristics during subjective perception and personalizing the automated vehicle following process for different types of drivers have become essential for evaluating advanced driver assistance systems to reduce the probability of traffic accidents^2^.

The longitudinal driver following model is mainly oriented to the driving scenario between adjacent vehicles in a single lane, for which various forms of following models have been established. For example, Bando et al. proposed the optimal velocity (OV) model, which assumes a relationship between the optimal vehicle velocity with the following distance and that the driver adjusts the velocity by acceleration and deceleration to achieve the optimal speed^3^. Khodayari et al. designed an artificial neural network to build the driver model, where the inputs are the estimated reaction time, following vehicle relative speed and distance, and main vehicle speed, and the output is the main vehicle acceleration and trained using the US NGSIM dataset^4^. A recurrent deep neural network has been used to build a microscopic driver following model that uses more historical states than instantaneous ones as inputs^5^. Zhou et al.^6^ used inverse reinforcement learning to model personalized driving behavior by learning historical data to build corresponding cost functions; Wang et al.^7^ analyzed multiple in-vehicle sensor parameters to classify driver following behavior into multiple decision states based on accelerator pedal operation and used support vector machines to build a driver behavior prediction model. Huang et al.^8^ built a following model based on long- and short-term memory neural networks to study the time lag phenomenon associated with asymmetric driving behavior and driver imperfect driving behavior. Zhang et al.^9^ used a nonlinear autoregressive neural network and a direct inverse model to learn the driver's actual vehicle following behavior, and completed a vehicle following model considering the driver's original driving style. The aforementioned following model can simulate different driving styles and predict different drivers' following behaviors by fitting the input feature parameters, but it is easy to ignore the state relationship between the internal feature parameters, and the key features need to be further explored.

In summary, most of the existing following models are built using experimental datasets of driving behavior from foreign countries, and these models reflect the following characteristics of foreign drivers. There are specific differences in traffic, vehicles, and driving styles and cultures in different countries and these differences are likely to result in significantly different driving behavior. Secondly, for the drivers, owing to the individual driver differences in reaction time and attention level as well as the strong subjectivity of their psychological characteristics, driving experience, and road environment perception^7^, the above methods, ignoring the state relationship between driver characteristic parameters and perception, are difficult to adapt to different drivers' driving habits and cannot effectively improve the satisfaction and acceptance of human–computer interaction and collaboration.

Based on the analysis of existing driver following behavior models, this paper proposes an adaptive longitudinal following model that integrates the perceptual process and driver characteristics. Unlike previous models, this model abstracts the influence of both external disturbance perception factors and driver characteristics on the following behavior into a mathematical expression. The model simulates the driver's subjective estimation of the driving environment under the perception of objective environmental disturbances, and is able to select the typical characteristic parameters of time distance and stopping distance to describe the driver's subjective ideal following distance, and use them to characterize the driver's following characteristics. The least recursive squares-based driver feature parameter identification algorithm is used to combine the driver following process to form an adaptive learning mechanism, and the identified parameters are used in the control module. Finally, the model performance of the established adaptive driver longitudinal following model and the simulation analysis of the difference of driver following behavior are carried out to verify the model performance in two dimensions: safety and following performance. The simulation of following behavior and subjective perception process based on driver characteristics in this model will provide some theoretical reference for the research of driver-assisted following system and active safety of automobiles.

## Methods

### Characteristics of the following behavior

To comprehensively consider the perception process and driving behavior differences, the adaptive driver following behavior model is constructed from the driver's perception, judgment, and operation process, mainly composed of driver perception, judgment and operation, feature parameter recognition, and vehicle modules. The key elements considered in the adaptive driver following model are the perception process of objective driving scenarios and reproduction of different types of driver behavior features, which are reflected in the model as the driver perception process and identification of driver longitudinal following behavior features.

#### Driver perception process description

Here, we simulate the driver's subjective perception of the driving environment. During vehicle following, the driver often estimates the front can’s speed and following distance based on the relative position change of the car in the field of view and size change of the front car’s rear, and this process has a certain independence and randomness^10^. From the above, if the accurate values of following distance and front vehicle speed obtained by sensors are directly input to the decision layer of the following model, the model results cannot reflect the following behavior of different types of drivers in real driving scenarios^11^. To obtain a model that matches drivers' driving behavior characteristics, the independence and randomness of perception must be considered during modeling. Hence, this module introduces the generalized Wiener process to describe the driver's perceptual characteristics. The resulting outputs will be used as the input to the judgment and operation module, which to some extent reflects the influence of the perception perturbed by the environment in the driver's following model.

#### Driver’s longitudinal following behavior feature recognition

Drivers have different expectations of following speed and driving distance when following another vehicle, and the time (in s) and stopping distances (in m) are usually chosen to describe the expected following distance^12^. Most of the current driver following models assume constant time and stopping distances, ignoring the differences in driver characteristics. Following models established in this way are less adaptable to drivers. Therefore, this study uses time distance and stopping distance as the characteristic parameters of driver following behavior, and they are divided into three categories to characterize aggressive, moderate, and conservative drivers, as shown in Table 1. The following data collected in the driver sensing module, including the vehicle's speed, speed of the vehicle in front, and following distance, indirectly represent the following style of the driver^13^. Thus, it is used as an input to the driver characteristic parameter identification algorithm, while the results of the identified driver characteristic parameters $\lambda$,$L$ are used to characterize the desired heel distance for this class of driver characteristics and are substituted into the design of the subsequent control module.

**Table 1:** Classification and values of driver characteristics parameters.

Driver type	$\lambda$(s)	$L$(m)
Radical	0.6 ~ 1.0	2.0 ~ 3.0
Moderate	1.1 ~ 1.5	3.0 ~ 4.0
Conservative	1.6 ~ 2.0	4.0 ~ 6.0

The following driving styles of different drivers vary greatly, i.e., drivers have different expectations of following speed and distance between cars during following, and the expected collision time distance $\lambda$ (unit: s) and expected stopping distance $L$ (unit: m) are usually selected to describe the subjective ideal expected following distance of drivers^12^. Where, $\lambda$ is the expected collision time distance (unit: s), which is mainly influenced by the driver's reaction time and attention level, and is generally taken as 0.6–2.0 s^12^; $L$ is the stopping distance (unit: m) judged by the driver, and is generally taken as 2–6 m^12^. Since many current studies of driver following models take the expected collision time distance $\lambda$ and expected stopping distance $L$ as constant values, ignoring the differences in driver characteristics, the following models established in this way are less adaptable to drivers. Therefore, in this paper, the expected collision time distance and expected stopping distance are used as the characteristics of driver following behavior, and they are equally divided into three categories to characterize the aggressive, moderate and conservative drivers, as shown in Table 1. The following data collected in the driver sensing module, including the speed of the vehicle, the speed of the vehicle in front, and the following distance, indirectly represent the following style of the driver^13^, so they are used as the input of the driver characteristic parameter identification algorithm, and the results of the identified driver characteristic parameters, the ideal desired following distance used to characterize this type of driver characteristic, are substituted into the design of the subsequent control module.

### Model structure

**Figure 1:**
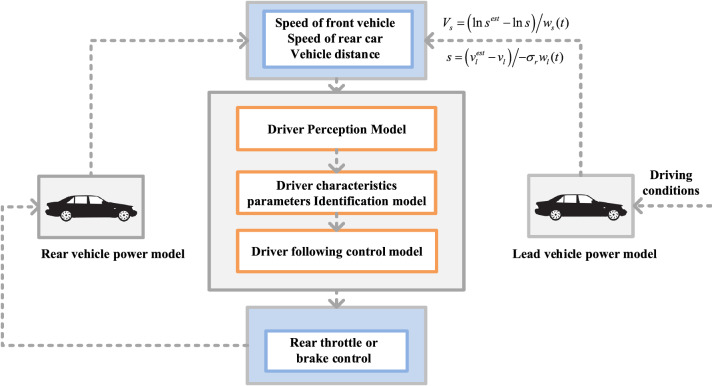
Example diagram of the driver's longitudinal following behavior model.

This study uses driver perception, judgment, and operational processes as the modeling process^14^. The model composition is shown in Fig. 1. In addition to building the models mentioned above, auxiliary evaluation models, including vehicle models, will be built as sub-models of the driver-following model.

#### Driver perception model construction

Here, the driver perception module uses the vehicle's speed ahead and following distance as inputs to build a driver perception model that simulates the driver's subjective driving environment. The model’s final output is the estimated speed and following distance of the vehicle ahead.

### Driver following distance perception process

In most driving situations, the relative error of the following distance can be considered constant, which is usually expressed as the logarithmic difference of the following distance, and the evolution of the driver's perception error of the following distance can be described as^14^

1 $$ \ln s^{est} - \ln s = V_{s} w_{s} (t), $$

where $V_{s}$ denotes the standard relative error of the logarithm of the workshop distance estimate $s^{est}$ and the actual value $s$, also known as the statistical coefficient of variation, assuming that the estimation error is unbiased, and $w_{s} (t)$ denotes a random variable following a standard normal distribution.

### Driver's front car speed perception process

**Figure 2:**
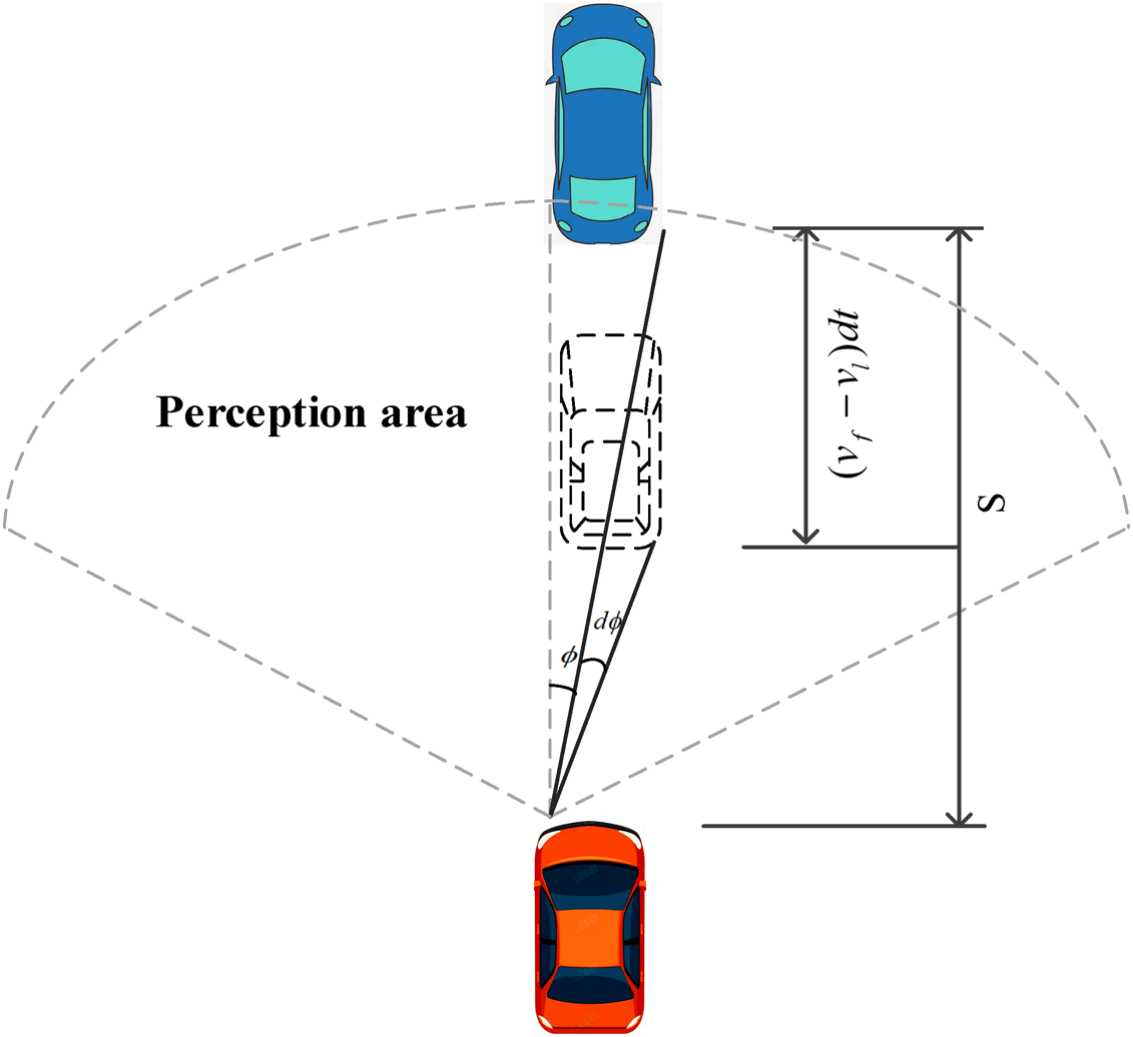
Relationship between driver's viewpoint change and relative speed.

During actual driving, the driver estimates the front car’s size by the change of its relative position and change in the rear size in the field of view^16^. In the top view describing the driver's field of view, the current view of the car in front $\phi$ is shown in Fig. 2, the change in view is $d\phi$, and the relative position of the front car in time $dt$ is $(v_{f} - v_{l} )dt$.

Based on the above reasoning, the rate of change of the visual angle $r$ is defined as follows.

2 $$ r = \frac{d\varphi /dt}{\varphi } = \frac{{\frac{{w_{veh} }}{{s^{2} }}\Delta v}}{{w_{veh} /s}} = \frac{\Delta v}{s} = \frac{1}{{\tau_{TTC} }} $$

Equation (2) shows that the relative viewpoint change rate $r$ is the reciprocal of the collision time (time-to-collision) $\tau_{TTC} = {s \mathord{\left/ {\vphantom {s {\Delta v}}} \right. \kern-\nulldelimiterspace} {\Delta v}}$, while $\tau_{TTC}$ is an important safety indicator to measure safety against collision. The front vehicle speed error is defined as follows.

3 $$ v_{l}^{est} - v_{l} = - (\Delta v^{est} - \Delta v) = - s\left( {\frac{1}{{\tau_{TTC}^{est} }} - \frac{1}{{\tau_{TTC} }}} \right) = - s\sigma_{r} w_{l} (t) $$

where $\sigma_{r}$ is the rate change of view standard deviation; similar to the random variables $w_{s} (t),\;w_{l} (t)$ describes the distribution of the estimation error of the forward speed and its variation over time. $w_{s} (t),\,\;w_{l} (t)$ are random variables obeying the standard normal distribution, reflecting the variation of the estimation error exhibited by different drivers under the combined factors of driving, psychological, and physiological conditions, and external environment perturbing the perception.

### Driver perceptual behavior characteristics simulation

The complexity of drivers' characteristics and external factors results in each driver having a mutually independent set of $\left\{ {w_{s} (t),\,w_{l} (t)} \right\}$, where the distribution function of the smooth stochastic process $w\left( t \right)$ is a standard Gaussian distribution function. If a driver underestimates the distance to a shop or the speed of the front car, it is likely that the driver will also underestimate the distance to the shop or the speed of the front car in the next few seconds, described mathematically as a correlation function, where two moments within a short time ($\tau$) of a few seconds to a minute are correlated^16^. Therefore, to simulate the change of the driver's perceptual error with the perceptual characteristics, the generalized Wiener process (Wiener process) of $w\left( t \right)$ is introduced to describe the driver's perceptual characteristics, which can be simulated by different initializations for different drivers and reflects the change in the driver's perceptual error for the speed and distance to the front car, whose stochastic differential equation is expressed as

4 $$ \sqrt {\frac{\tau }{2}} \frac{dw}{{dt}} + \sqrt {\frac{1}{2\tau }} w = \xi (t) $$

The driver estimation error time $\tau$ is the model parameter and $\xi \left( t \right)$ is the standardized white noise (standardized white noise), and the solution of the stochastic differential Eq. (4) can be expressed as

5 $$ w(t) = \frac{1}{{a_{0} }}\int_{{t_{0} }}^{t} {e^{a(t - s)} \xi (s)ds = \sqrt {\frac{2}{\tau }} \int_{0}^{t} {e^{{ - \frac{t - s}{\tau }}} } \xi (s)ds} $$

Two sets of pseudo-random numbers initialized with white noise are chosen and substituted into Eq. (5) to solve a set of mutually independent $\left\{ {w_{s} (t),\,w_{l} (t)} \right\}$ to represent the perceptual characteristics of the driver under the perturbed conditions.

According to Eqs. (1) and (3), the driver's perceived speed and following distance {$v_{l}^{est}$, $s^{est}$ } can be obtained, and the results will be used as the input of the driver's judgment and operation model, which can simulate the influence of the driver's perception in the driver following model to a certain extent. This study mainly considers the driver's perception of the front vehicle’s speed and following distance. According to the above derivation, the relevant parameters of the driver perception model are shown in Table 2.

**Table 2:** Driver perception model-related parameters.

Perceptual model parameters	Reference values
Workshop distance estimation error	10%
Standard deviation of the rate of change of angle of view	0.01 s^-1^
Estimated error duration	20 s

#### Longitudinal following control model for adaptive identification of driver behavior characteristic parameters

The adaptive driver characteristic parameter identification algorithm is based on the recursive least squares (RLS) algorithm^17^. During recursive parameter estimation, each new observation is used to correct the previous estimation according to the recursive algorithm to recursively obtain new parameter estimates.

Driver following behavior parameter identification aims to identify the driver parameters $\lambda$ , $L$ based on the following distance $s^{est} (t)$ in $\Delta T$ time and the autospeed $v_{f} (t)$ . The identified parameters can be used as input parameters for the driver longitudinal following behavior model to establish a following behavior model based on the perceptual process and driver behavior features. The recursive least squares algorithm used in the adaptive parameter identification algorithm is as follows:

The parameter vector $\hat{\user2{\theta }}(k)$ is represented by.

6 $$\hat{\user2{\theta }}(k) = [\lambda (k)\;\;L(k)]^{T}$$

where $\lambda (k)$ and $L(k)$ are the discrete values of Expected collision time distance and expected stopping distance, respectively, and $k = 0,\;1,\;2,\; \ldots ,\;N$, $N$ are positive integers, and the input vector $\hat{\user2{h}}(k)$ by

7 $$ \hat{\user2{h}}(k) = [v_{f} (k)\;\;1]^{T} , $$

where $v_{f} (k)$ is the discrete value of the self-tracking speed.

The output variable $\hat{z}(k)$ is expressed as

8 $$ \hat{z}(k) = \hat{\user2{h}}^{T} (k)\hat{\user2{\theta }}(k) $$

where $\hat{z}(k)$ is the discrete following distance.

**Figure 3:**
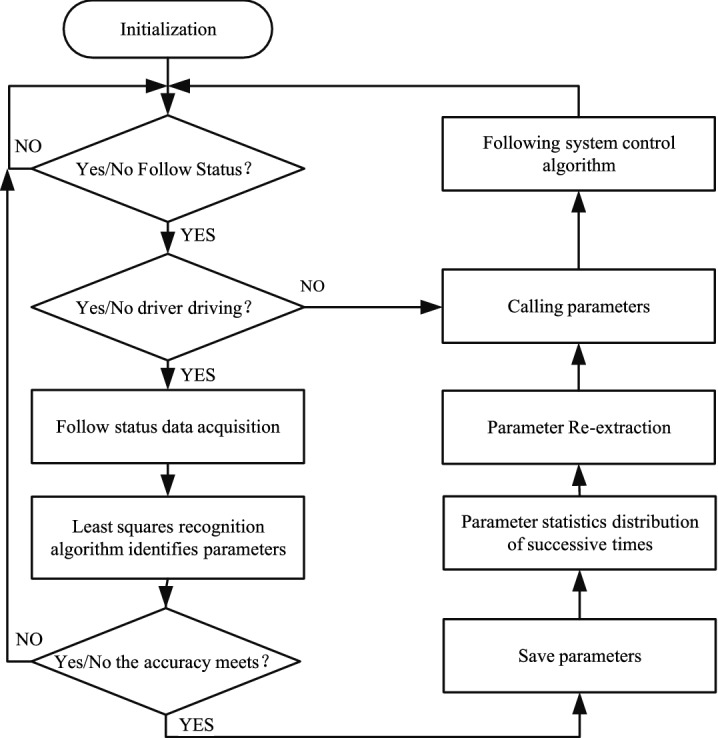
Flow chart of self-learning parameter recognition algorithm for driver characteristics.

The adaptive following model will collect driver following data when a driver fully controls a vehicle. The data will be used as input to the driver characteristic parameter identification algorithm; When the driver is not in full control, the driver characteristic parameters identified after the final recorded data is called. The flow chart of the adaptive driver characteristic parameter recognition algorithm is shown in Fig. 3.

**Figure 4:**
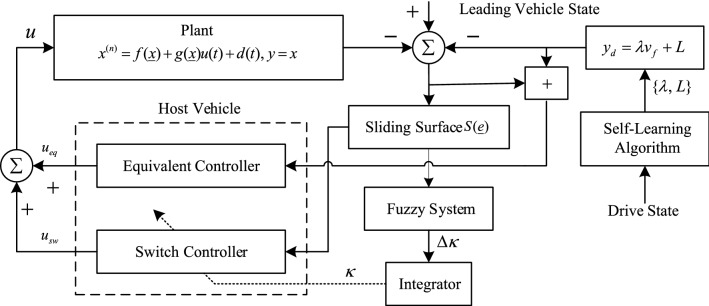
Block diagram of the slip model control system for a driver longitudinal following behavior model.

According to the driver following behavior identified by the feature recognition algorithm, the identified driver feature parameters $\lambda$ and $L$ are used to determine the ideal following distance for different types of drivers $s_{d}$. In this study, a sliding mode variable structure control algorithm is used to approximate the ideal following distance^16^, and the saturation function replaces the symbolic function and compensates its boundary layer with fuzzy inference to partially eliminate the jitter^13^ to establish a driver following model based on the identification of driver characteristic parameters. In this study, the system block diagram of the driver feature-based following control algorithm is designed as shown in Fig. 4.

The input control quantity $u$ ($u > 0$ for throttle input and $u < 0$ for brake input) in the driver longitudinal following kinematic state equation corresponds to the driver's operation of the vehicle's throttle and brake pedal, which visually represents the driver's operation state^18^; the output of the state equation is the distance, speed, and acceleration of the vehicle. The driver longitudinal following kinematic state equation provides an important theoretical basis for establishing the driver longitudinal following behavior model. The constructed vehicle longitudinal kinematic state equation is

9 $$ \left\{ \begin{gathered} \dddot x_{f} = f({\varvec{X}}) + g({\varvec{X}})u(t) + d(t) \hfill \\ u = \frac{1}{{g({\varvec{X}})}}\left( {c_{1} \dot{e} + c_{2} \ddot{e} + \dddot x_{l} - \lambda x_{f}^{(4)} - f({\varvec{X}}) + \hat{\kappa } \cdot sat(S({\varvec{X}}))} \right) \hfill \\ \end{gathered} \right. $$

The system outputs are the following distance $y = x_{f}$,${\varvec{X}} = [x_{f} ,\dot{x}_{f} ,\ddot{x}_{f} ]^{T}$,$\dot{x}_{f}$, and the following speed of the vehicle $\ddot{x}_{f}$, which can be obtained from the system outputs. The kinematic physical quantities of the following distance, speed, and acceleration can be obtained from the output of the following model. The derived quantities of the following distance, relative speed, and acceleration difference can be obtained according to the previous vehicle's motion state. These physical quantities describe the driver's following behavior from the perspective of describing the vehicle motion state, enabling the simulation of the driver's longitudinal following behavior model.

#### Model evaluation scheme

This study investigates the elements of a driver following behavior model, mainly containing two aspects: driver characteristics and model evaluation elements.

First, the driver characteristic elements are reflected in drive following modeling, that is, the determination of the ideal driver following distance, usually determined by Expected collision time distance and expected stopping distance^19^; hence, this study uses both as driver characteristic parameters to consider the influence of driver characteristic differences in the longitudinal following behavior model and improve the model’s adaptability to different driver characteristic parameters.

The safety and following are the second model evaluation elements that primarily guarantee safety. These elements ensure that no vehicle collision occurs during following, and the reciprocal of the time-to-collision $\tau_{{TTC^{ - 1} }}$ is used as the index; the following element, which improves road utilization and ensures that the vehicle drives according to the ideal following distance, also describes the driver's control of the vehicle in maintaining the target following distance and speed. The signal-to-deviation ration (SDR), mean error (ME), mean absolute error (MAE), mean absolute relative error (MARE), and root mean square error (RMSE) of the actual and target following distances are used as the indexes to judge the following performance^20^. Comfort assessment is based on the premise of safe following, and comfort is expressed as the driver does not experience discomfort caused by violent acceleration changes during vehicle acceleration or deceleration. The acceleration rate of change $j_{c} = \dot{a}_{f}$ as the comfort evaluation index is the common method, usually the acceleration rate of change corresponds to the comfort range of $- 2 \le j_{c} \le 2$.This study develops safety and following assessment models for the above-analyzed elements of the driver's longitudinal following behavior model, and they are used to demonstrate the reliability and accuracy of the adaptive following model^21^.

## Results

The adaptive following model is designed to simulate the driver’s following process. The driver’s decisions are often after sensing external information, and the brain’s judgment is expressed as the control of the vehicle^22^. Therefore, this study discusses the driver’s following behavior from the perspective of vehicle control and the vehicle’s motion state and verifies the functioning of the constructed adaptive longitudinal following model that integrates the sensing process and driving characteristics.

### Verification of the basic function of the follower model

**Figure 5:**
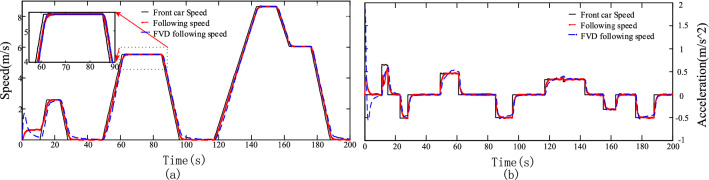
(**a**) Simulation results of the following speed curve for Case 1. (**b**) Simulation results of the following acceleration curve for Case 1.

**Figure 6:**
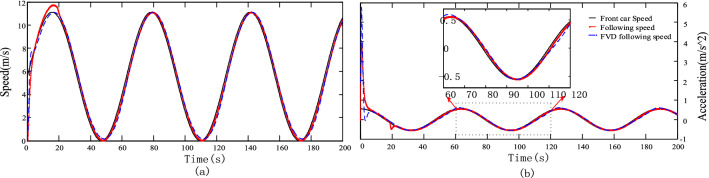
(**a**) Simulation results of the following speed curve for Case 2. (**b**) Simulation results of the following acceleration curve for Case 2.

We selected two sets of common working conditions, namely the standard ECE working (working condition 1) and sinusoidal working conditions (working condition 2), as test conditions to simulate the speed profile of the front vehicle, whose horizontal and vertical axes are the time and speed, respectively. Secondly, the optimized speed FVD model is mostly used in the study of nonlinear characteristics of traffic flow under multi-vehicle following, two-lane following and lateral interference. Based on its characteristic of mainly considering the driver's desired speed, the driver's own driving habits and environmental interference are more relevant to the model results, and the FVD model is selected as the control group of this paper's model.Assuming an initial vehicle separation of 10 m, the simulation tests of the FVD model and the proposed model are conducted under the two conditions. The following speed and distance and acceleration simulation results of the two models for working conditions 1 and 2 are shown in Figs. 5 and 6, respectively.

The FVD model responds quicker and has greater acceleration when approaching the front car, as shown in Fig. 6. The acceleration curve corresponding to working condition 2, the FVD following acceleration using the proposed following acceleration model is $5.8\;{\text{m}}/{\text{s}}^{2}$, which is smaller than the former $3.9\,{\text{m}}/{\text{s}}^{2}$. Meanwhile, Fig. 5 shows the acceleration curve corresponding to working condition 1, before the front car starts, for the acceleration of the following car. In the following process, except for the uniform speed and stationary stages, the proposed model responds to the speed of the front car faster, and the speed curve only lags behind the speed of the front car, while the speed curve of the FVD model lags behind the speed curve of the proposed model, indicating that when the front car accelerates, the following car follows the acceleration curve. When the front car accelerates, the following car also accelerates toward the speed of the front car; when the front car has a constant speed, the following car retains the peed of the front car to maintain the ideal following distance; when the front car decelerates, the following car also decelerates; within the safety range, the following car is not expected to decelerate sharply to reach the speed of the front car, and the speed change of the following car is always slightly delayed compared with that of the front car. The speed and acceleration curves of the proposed and FVD models meet the vehicle's speed and acceleration change characteristics when following the vehicle and realize the speed and acceleration following function.

The change in car separation during the following process is the external expression of the driver's following behavior. This model adopts the ideal following distance determined by the driver's characteristic parameters for the actual following distance to be close to the ideal following distance. Therefore, the proposed model’s following distance is closer to the ideal following distance. The following distance simulation curves for working conditions 1 and 2, and the following distance corresponding to both models is greater than the safety distance, that is, when the front vehicle suddenly decelerates sharply, the following car can brake within a limited time to prevent a collision; secondly, the following distance of this model performs well in the approximation of the ideal distance.

### Validation and analysis of the following speed under different driving behavior characteristics

**Figure 7:**
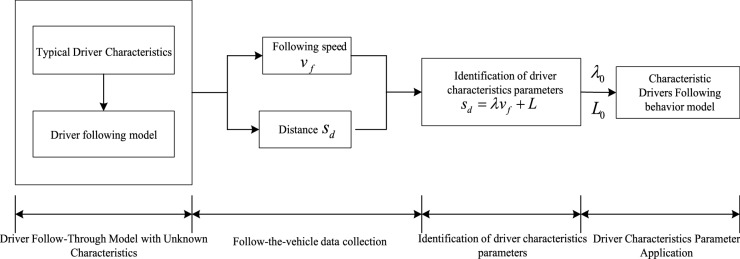
Driver characteristic parameter identification test program flow.

In this study, the three typical driver characteristic parameters in Table 1, that is, aggressive, moderate, and conservative, represent the drivers, A, B, and C, respectively. The driver characteristic parameter identification test scheme is shown in Fig. 7.

**Figure 8:**
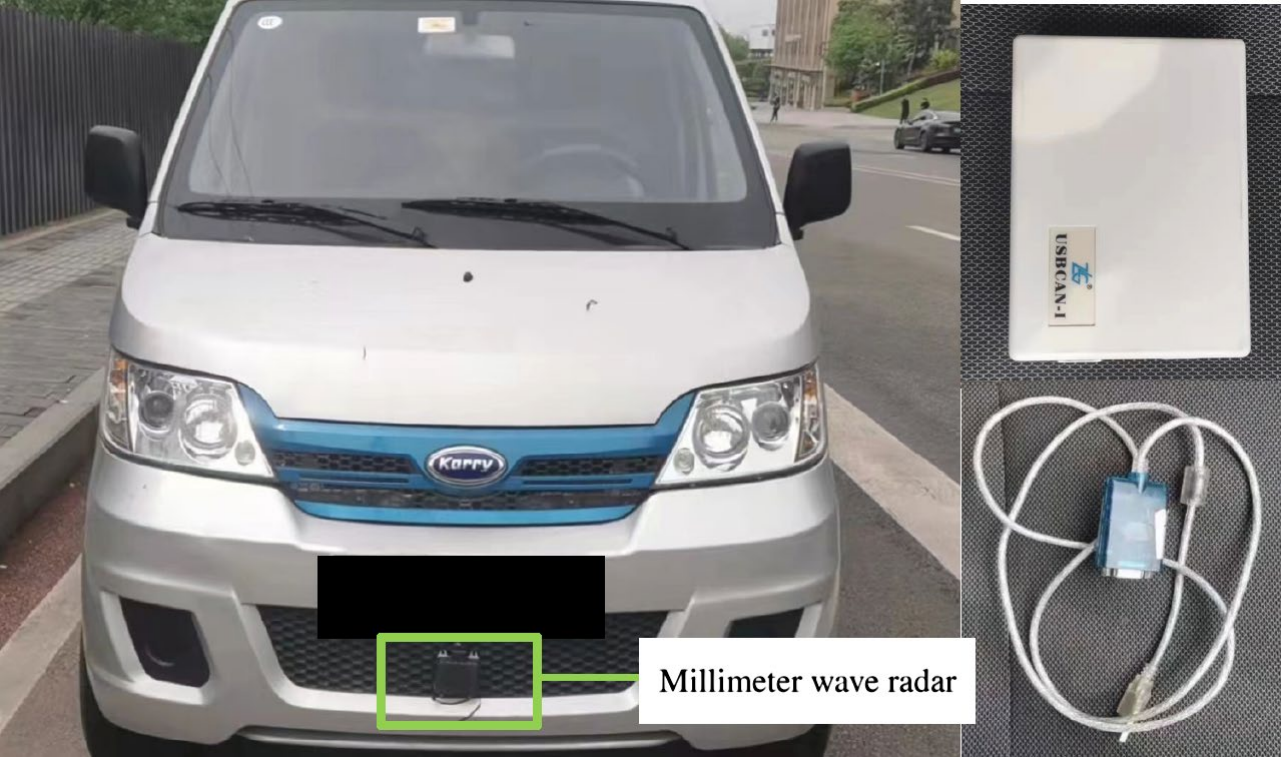
Millimeter wave radar installation on experimental vehicle.

**Figure 9:**
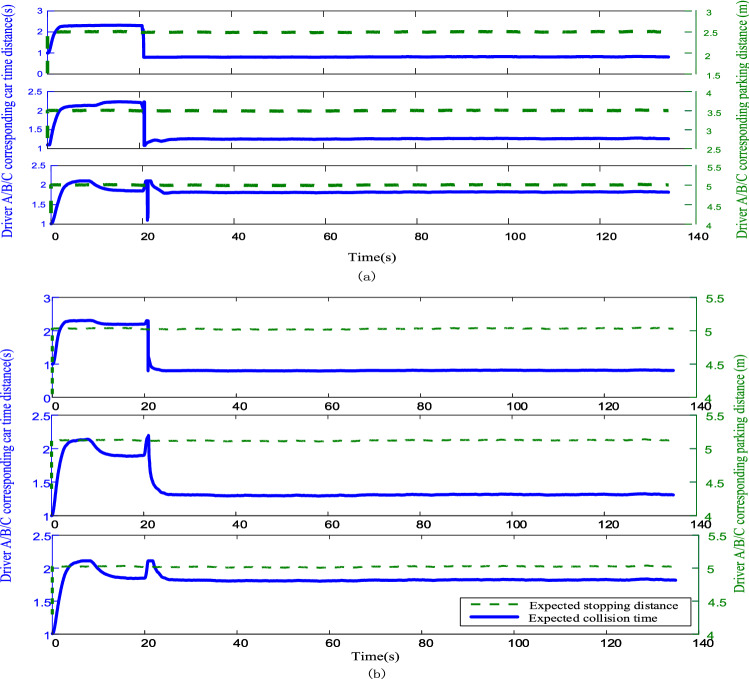
(**a**) Identification curves of three types of driver characteristics based on this model (**b**) Identification curves of three types of driver characteristics based on the control group model.

According to the test protocol, multiple real-world driving data collection was performed, and the equipment required for the experiment is shown in Fig. 8^6^. The three groups of following distance and speed data with a duration time series length of 135 s were selected as the input for the identification model of the characteristic parameters and the control group model^9^ considering individualized driving style, and the three groups of data represent the following data of aggressive, moderate, and conservative A, B, and C drivers, respectively, and the identification curves of the three types of driver characteristic parameters can be obtained after the driver characteristic parameter identification model. as shown in Fig. 9.

In Fig. 9, the initial car separation is greater than the ideal following distance, giving the three types of driver characteristic curves the same trend initially, and the maximum time distance is more than 2 s. When the time exceeds 20 s, the driver characteristic curve smoothens. Compared with the two models, the model in this paper has a flatter change, no large jumps, and more continuity. The three sets of curves from top to bottom represent the aggressive, moderate, and conservative drivers, where the parameters characterizing aggressive driver following behavior show short time and stopping distances, fluctuating slightly around 0.8 s and 2.5 m, respectively, while the parameters characterizing conservative driver following behavior show larger time and stopping distances, fluctuating slightly around 1.8 s and 5.0 m, respectively. The conservative driver following behavior parameters show large time and stopping distances with small fluctuations around 1.8 s and 5.0 m, respectively, while the parameters characterizing the following behavior of moderate drivers are in between, with slight fluctuations around 1.25 s and 3.5 m, respectively.

**Figure 10:**
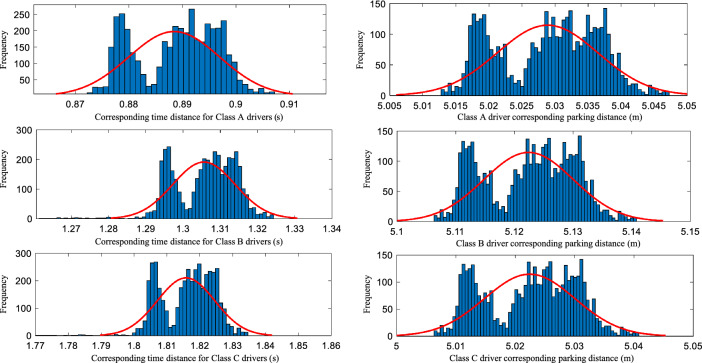
Frequency histograms of the feature parameters corresponding to the three types of feature drivers.

The data corresponding to the characteristic parameters of drivers A, B, and C were statistically analyzed to obtain the frequency histograms of the characteristic parameters for the three types of characteristic drivers, as shown in Fig. 10. The expectation and correlation coefficients were obtained for the three groups of identified driver characteristic parameters, and the results of the characteristic parameter identification were obtained as shown in Table 3. According to the identification results of the driver characteristics, the errors between the identified expectation and target values of the three characteristic groups are within 0.1%, indicating that the identified expectation values have good correlation, which can be used to characterize aggressive, moderate, and conservative driver following behavior.

**Table 3:** Driver characteristics parameters identification results.

Serial number	Characteristic parameters	Target value	Expected value of the identification value	Error (%)	Correlation coefficient
1/A	$\lambda$	0.82	0.8256	0.06	0.9995
	$L$	2.50	2.4989	0.04	
2/B	$\lambda$	1.26	1.2613	0.10	0.9994
	$L$	3.50	3.5024	0.06	
3/C	$\lambda$	1.82	1.8194	0.03	0.9997
	$L$	5.00	5.0024	0.05	

^1^The serial number 1/A indicates the first group of feature target values, group A feature recognition expectation values, etc.

To determine the influence of the driver characteristic parameters on the driver following model, the identification algorithm was used to identify the last three groups of typical characteristic parameters as the model parameters of the following control system. Two standard operating conditions, ECE and FTP75 (U.S. urban cycle operating conditions), were introduced as test conditions^23^ to verify the drivers’ longitudinal following behavior for different driver characteristic parameters under the same operating conditions. They were also used to analyze and compare the variability of the adaptive model in processing different driver following data and evaluating and analyzing the reproduced types of driver following behavior according to the evaluation index^22^.

#### Analysis of the variability of the Follower model

**Figure 11:**
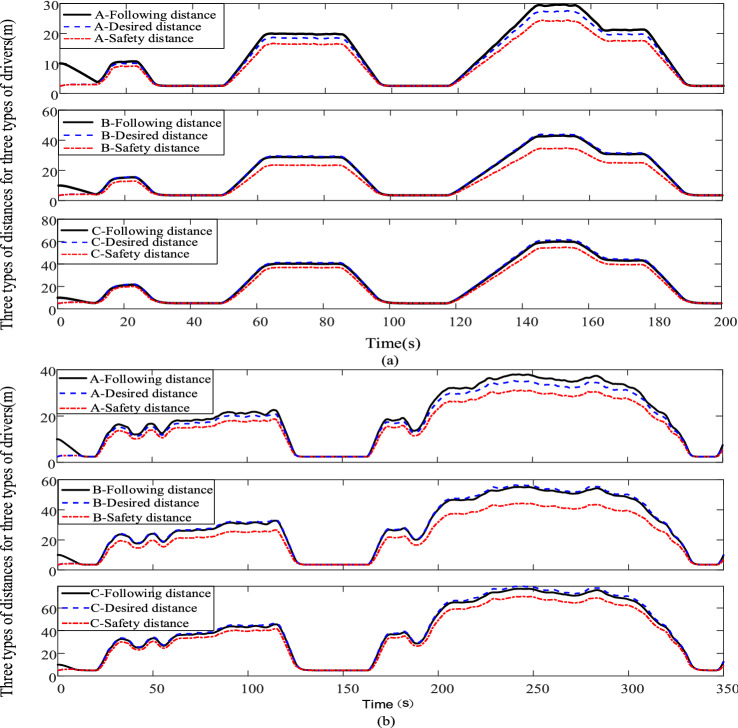
(**a**) Three types of driver following, ideal, and safety distances for standard operating conditions ECE. (**b**) Three types of driver following, ideal, and safety distances for the standard operating condition FTP75.

The driver following behavior simulation results with three types of characteristics corresponding to the working conditions ECE and FTP75 are shown in Fig. 11.

As shown in Fig. 11a and b, according to the comparison of the curves of the following distance and spacing error for three types of drivers, aggressive A, moderate B, and conservative C, under the same working conditions. Aggressive driver A is expected to maintain a smaller following distance, while conservative driver C is expected to maintain a larger following distance, and moderate driver B is expected to maintain a following distance between the first two. The following distances for all three types of drivers are close to the ideal following distance and greater than the safe following distance, indicating that the driver has sufficient reaction time to brake and avoid a collision. The following distance expectations of the three driver categories reflect the differences in their desired ideal following distances and characteristics of different driver following styles.

#### Safety analysis of the follower model

**Figure 12:**
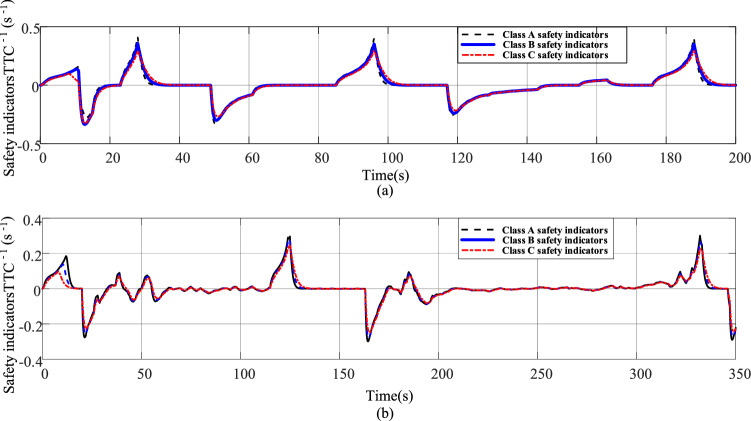
(**a**) The three types of driver following safety indicators for the standard working condition ECE. (**b**) Three types of driver following safety indicators corresponding to the standard working condition FTP75.

The simulation results of the following safety indicators for the three characteristic drivers for working conditions ECE and FTP75 are shown in Fig. 12.

As shown in Fig. 12a and b, the actual following distance of all three types of characteristic drivers is greater than the critical safe following distance, allowing the drivers to brake to ensure safety within the limited reaction time; The comparison of the safety index curves shows the corresponding $TTC^{ - 1}$ curves of the working conditions ECE and FTP75, and deceleration of all characteristic drivers when the front car decelerates between [− 2.2, 0] (unit: ${\text{m}}/{\text{s}}^{2}$), and the corresponding safety indicators are all included in [0, 0.5] (unit: ${\text{s}}^{ - 1}$), which are within the effective safety range of the safety evaluation indicators shown in Table 4; when the front car accelerates, if it is assumed that it may suddenly decelerate at the current acceleration moment, as shown in Fig. 12a and b, the safety indicators are still within the effective safety range.

**Table 4:** Range of safety indicators ($s^{ - 1}$)^24^.

Percentage /$\tau_{{TTC^{ - 1} }}$	5%	Average value	95%
Acceleration (${\text{m}}/{\text{s}}^{2}$)			
$- 2 < a < - 0.5$	− 0.02	0.11	0.36
$- 4 < a < - 2$	− 0.01	0.25	0.60
$- 6 < a < - 4$	0.23	0.70	1.50
$a < - 6$	0.56	1.13	1.72

#### Followership analysis of the followership model

**Figure 13:**
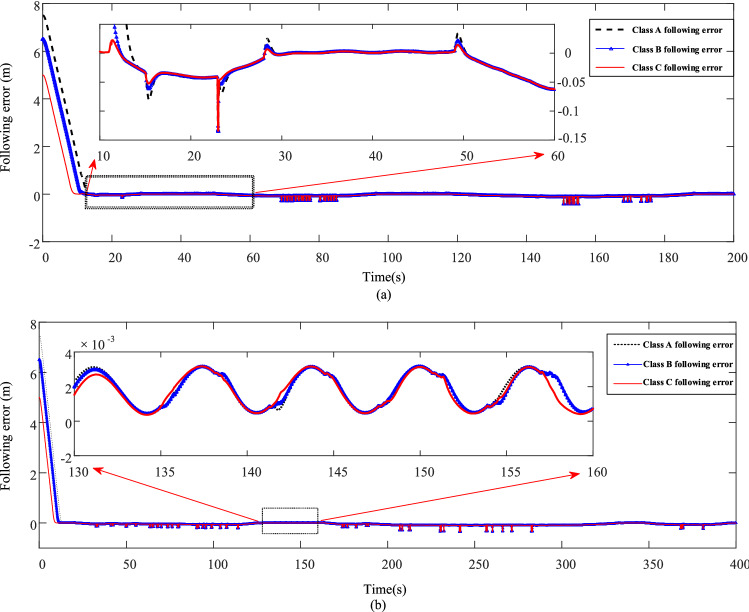
(**a**) Three types of driver following errors corresponding to the standard working condition ECE. (**b**) Three types of driver following errors corresponding to the standard working condition FTP75.

The simulation results of the following error for the three types of characteristic drivers corresponding to working conditions ECE and FTP75 are shown in Fig. 13.

A comparison of the curves in Fig. 13a and bshows that the following errors of drivers A, B, and C, excluding the effect of stopping distance, decrease from 7.5 m, 6.5 m, and 5.0 m, respectively, due to the initial vehicle distance of 10 m. The final error range remains between [− 0.2 m, 0.05 m] and [− 0.002 m, 0.002 m] for the ECE and FTP75 conditions^25^. Under the 10 m initial distance, drivers A, B, and C each believe that there is 7.5 m, 6.5 m, and 5 m to the front car. This difference indicates the following behavior of the characteristic drivers for the following distance. Tables 5 and 6 show the following indicators of the characteristic drivers for the ECE and FTP75 working conditions.

**Table 5:** Evaluation indexes of three types of driver following behavior models corresponding to ECE working conditions.

Indicators	Follow-through				
Drivers	SDR	ME	MAE	MARE	RMSE
A	5.176	− 0.127	0.1274	0.0168	0.5919
B	11.532	− 0.0082	0.0394	0.0067	0.3574
C	15.669	− 0.0004	0.0314	0.0034	0.2619

**Table 6:** FTP75 working conditions corresponding to the three types of driver following behavior model evaluation index.

Indicators	Follow-through				
Drivers	SDR	ME	MAE	MARE	RMSE
A	6.549	− 1.5573	1.5573	0.1404	2.0218
B	12.395	0.9316	1.1816	0.0654	1.5035
C	12.613	0.4795	0.8302	0.0451	1.3019

As shown in Tables 5 and 6, the following index SDR indicates the signal deviation rate between the actual and ideal following distances, and a larger SDR indicates a greater approximation of the actual following distance to the ideal following distance^26^, which shows that the following distance of the aggressive driver is the least like its ideal following distance, and the following distance of the conservative driver has the largest SDR, while that of the moderate driver is between the two, as verified by other following indicators, such as ME, MAE, and MARE^27^. Aggressive drivers have the quickest reaction according to the motion state change of the front vehicle and most timely acceleration or deceleration with the largest acceleration or deceleration amplitude. They also have more than expected operations in a shorter reaction time, making the control of the degree of approximation to the ideal following distance more difficult. Since the conservative driver maintains a longer following distance, it is easier to control the vehicle to drive according to the expected motion in a longer reaction time. The control for the moderate driver is in between, and the following index is more similar to the conservative driver's index, and can control the vehicle better in a shorter reaction time.

#### Comfort analysis of vehicle following model

**Figure 14:**
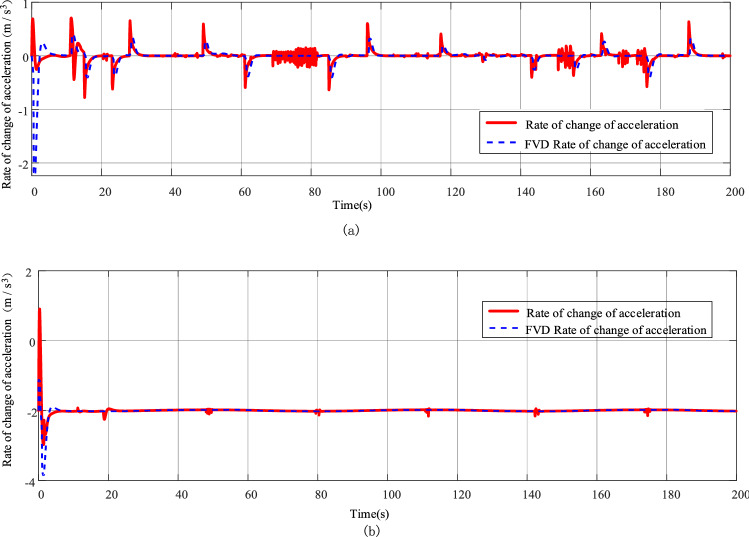
(**a**) Simulation results of the rate of change of following acceleration corresponding to working condition 1. (**b**) Simulation results of the rate of change of following acceleration corresponding to working condition 2.

The simulation results of the rate of change of following acceleration for the two models corresponding to working condition 1 and working condition 2 are shown in Fig. 14.

The acceleration rate of change $j_{c} = \dot{a}_{f}$ is a common method to evaluate the comfort level, and the comfort range of the acceleration rate of change is usually in $- 2 \le j_{c} \le 2$. The acceleration rate of change curve corresponding to working condition 1, as shown in Fig. 14a, the FVD model has acceleration rate of change exceeding $- 2m/s^{3}$ in the beginning of 0 ~ 3 s, while the present model does not have a moment out of range; the acceleration rate of change curve corresponding to working condition 2, as shown in Fig. 14b, both the FVD model and the present model have acceleration rate of change exceeding $- 4\,{\text{m}}/{\text{s}}^{3}$ in the beginning of 0 ~ 3 s, while the present model has only one acceleration rate of change exceeding D in the beginning of 0 ~ 3 s. The FVD model and the present model both have acceleration changes exceeding D within 0 ~ 3 s at the beginning, while the present model has only one acceleration change rate critical $- 2\,{\text{m}}/{\text{s}}^{3}$ moment. It can be seen that this model performs better than the FVD model in terms of comfort following.

## Discussion

Optimizing driver following behavior, which must be mastered by intelligent transportation systems for active vehicle safety, is important for improving traffic efficiency and safety and other needs. In summary, in order to verify the longitudinal following behavior model established in this paper, the classical driver following model FVD model is introduced, and the comparison results show that this model can better simulate the driver following behavior, and the following index is relatively improved by 80%, while the following control simulated in this model is more comfortable on the basis of safety. Secondly, this paper uses three sets of identified driver characteristic parameters to simulate different driver following behavior characteristics, which are aggressive, conservative and moderate. From the comparison results, it can be seen that the aggressive driver reacts more quickly to following, the conservative type reacts relatively slowly, and the moderate type is moderate, while the conservative driver has the best following and comfort, the aggressive type has the worst performance, and the moderate type is in the middle of the two, and the following is relatively improved by 55% and 36% compared with the aggressive type. driver behavior with different characteristic parameters.

1. Personalized driver following models must adapt to different drivers' desired following distance and behavior, establish an intrinsic link between the external environment and subjective driver behavioral characteristics, and introduce model the desired following distance parameters. The model learns driver behavior through the least recursive squares method. The model is highly explanatory and easily describes the uncertainty of driving behavior. It can provide a more accurate, anthropomorphic, and driver-expected driver following model for adaptive cruise control and automatic following systems of intelligent vehicles.
2. Validation with natural driving behavior experimental data shows that the proposed adaptive following model, integrating perception and driver behavior, shows good performance in describing individual driver following behavior. The comparison with the prediction results of the classical FVD model shows that the average error calculated of the described model is 80% higher in absolute terms, which is in better agreement with the actual following behavior of the driver.
3. To make the proposed model more robust and adapatable, the number of drivers tested and diversity of samples will be increased by considering special scenarios such as bystander car cut-in and emergency braking of the front vehicle.

The adaptive driver following model studied in this paper is designed for the independence of driver perception error, randomness and their own driving behavior characteristics, which can better fit the longitudinal following behavior of different drivers in real driving scenarios. The next work can be combined with practical experiments to enhance the reliability of the control model.

## Data Availability

The data used in this study are publicly available.

## Code availability

The code that supports the findings of this study are available from the corresponding author upon request.
